# Monthly Record of Deaths in the City of Buffalo

**Published:** 1859-05

**Authors:** P. H. Strong

**Affiliations:** Health Physician


ART. V.—*Report of Mortality in Buffalo for the Month of March*, 1859. By P. H. STRONG, M. D., Health Physician.

| DISEASES. | | No. | Males. | Females. | No Sex given. |
|---|---|---|---|---|---|
| Accidental, | | | | | |
| By fall in Hold of Vessel, | 1 | | | | |
| By Drowning, | 2 | | | | |
| By Locomotive, | 2 | 5 | 4 | 1 | |
| Anæmia, | | 1 | 1 | | |
| Asphyxia, | | 1 | 1 | | |
| Asthma, | | 1 | | 1 | |
| Cancer of Womb, | | 2 | | 2 | |
| Cholera Infantum, | | 2 | 2 | | |
| Congestion of Brain, | | 2 | 2 | | |
| Congestion of Lungs, | | 3 | 1 | 1 | 1 |
| Convulsions, | | 7 | 7 | | |
| Croup, | | 5 | 2 | 3 | |
| Debility, Infantile, | | 10 | 5 | 5 | |
| Delirium Tremens, | | 2 | 1 | 1 | |
| Dentition, difficult, | | 1 | 1 | | |
| Diarrhœa, | | 1 | 1 | | |
| Disease of Heart, | | 1 | 1 | | |
| " Lungs, | | 1 | 1 | | |
| Dropsy of Abdomen, | | 1 | | 1 | |
| Dropsy of Brain, | | 4 | 2 | 2 | |
| Erysipelas, | | 1 | 1 | | |
| Exhaustion, | | 1 | 1 | | |
| Fever, Puerperal, | | 3 | | 3 | |
| " Scarlet, | | 7 | 3 | 4 | |
| " Typhus, | | 1 | 1 | | |
| " Typhoid, | | 2 | | 2 | |
| Hooping Cough, | | 1 | | 1 | |
| Inflammation of Brain, | | 4 | 3 | 1 | |
| " of Bowels, | | 1 | | | 1 |
| " of Lungs, | | 7 | 4 | 3 | |
| " of Liver, | | 1 | 1 | | |
| Intemperance, | | 2 | 2 | | |
| Marasmus, | | 3 | 3 | | |
| Old Age, | | 4 | 2 | 2 | |
| Rheumatism, Chronic, | | 1 | 1 | | |
| Small Pox, | | 8 | 5 | 2 | 1 |
| Still Born, | | 10 | 8 | 2 | |
| Tuberculosis, | | 18 | 9 | 9 | |
| Uterine Hæmorrhage, | | 1 | | 1 | |
| Uterine Tumor, | | 1 | | 1 | |
| Unknown, | | 6 | 2 | 4 | 3 |
| Total, | | 133 | 77 | 53 | |

### SEXES.

| | |
|---|---|
| Males, | 77 |
| Females, | 53 |
| Sex not given, | 3 |
| Total, | 133 |

| | | |
|---|---|---|
| Died at the Almshouse, | 4 | |
| " Hospital of the Sisters of Charity, | 5 | |
| " Foundling Asylum, | 2 | |
| " Small-pox Hospital, | 1 | 12 |
| " in city at large, | | 121 |
| Total, | | 133 |

| | |
|---|---|
| Of this No. there were certified by Undertakers, | 40 |
| " " " by Coroner, | 8 |
| " " " by Irregular Practitioners, | 26 |
| " " " by Regular Physicians in Public Institutions, | 12 |
| " " " by Regular Physicians in Private Practice, | 36 |
| " " " by the "*Spirit of John Sweny,*" | 1 |
| Total, | 133 |

### AGES.

| | | | |
|---|---|---|---|
| Still-born, | 10 | Between 40 " " 50 " | 8 |
| Under 1 year | 36 | " 50 " " 60 " | 5 |
| Between 1 year and 5 years, | 24 | " 60 " " 70 " | 6 |
| " 5 " " 10 " | 7 | " 70 " " 80 " | 3 |
| " 10 " " 15 " | 2 | " 80 " " 90 " | 1 |
| " 15 " " 20 " | 5 | " 90 " " 100 " | 0 |
| " 20 years and 30 years, | 14 | " 100 " | 0 |
| " 30 " " 40 " | 7 | Unknown, | 4 |

| | |
|---|---|
| Total less than 5 years, | 70 |
| Total at 5 years and over, and unknown, | 63 |
| Total, | 133 |

### NATIVITIES.

| | | | |
|---|---|---|---|
| American, | 95 | French, | 3 |
| German, | 10 | Holland, | 0 |
| Irish, | 16 | Swiss, | 1 |
| Canadian, | 0 | Prussian, | 0 |
| English, | 3 | Italian, | 0 |
| Scotch, | 2 | Country not given, | 3 |

| | |
|---|---|
| Total, | 133 |

Comparative March mortality for the 5 years next preceding 1859 :

| | | |
|---|---|---|
| For March, 1854, | 181 | |
| " 1855, | 123 | |
| " 1856, | 111 | |
| " 1857, | 235 | |
| " 1858, | 134 | |
| Mean March mortality for 5 years, within a fraction, | | 137 |
| For March this year, | | 133 |

